# The immunological response to syphilis differs by HIV status; a prospective observational cohort study

**DOI:** 10.1186/s12879-017-2201-7

**Published:** 2017-01-31

**Authors:** Chris Kenyon, Kara Krista Osbak, Tania Crucitti, Luc Kestens

**Affiliations:** 1 HIV/STI Unit, Institute of Tropical Medicine, Antwerp, Belgium; 2 Division of Infectious Diseases and HIV Medicine, University of Cape Town, Anzio Road, Observatory 7700, Cape Town, South Africa; 3 HIV/STI Reference Laboratory, Institute of Tropical Medicine, Antwerp, Belgium; 4 Immunology Unit, Institute of Tropical Medicine,, Antwerp, Belgium; 5 Department of Biomedical Sciences, University of Antwerp, Antwerp, Belgium

**Keywords:** Syphilis, HIV, Immunology, IL-10, *Treponema pallidum*

## Abstract

**Background:**

It is not known if there is a difference in the immune response to syphilis between HIV-infected and uninfected individuals.

**Methods:**

We prospectively recruited all patients with a new diagnosis of syphilis and tested their plasma for IFNα, IFNγ, IL-1β, IL-12p40, IL-12p70, IP-10, MCP-1, MIP-1α, MIP-1β, IL-4, IL-5, IL-6, IL-7, IL-8, IL-10 and IL-17A at baseline pre-treatment and 6 months following therapy.

**Results:**

A total of 79 HIV-infected [44 primary/secondary syphilis (PSS) and 35 latent syphilis (LS)] and 12 HIV-uninfected (10 PSS and 2 LS) cases of syphilis and 30 HIV-infected controls were included in the study. At the baseline visit, compared to the control group, concentrations of IL-10 were significantly elevated in the HIV-infected and uninfected groups. The level of IL-10 was significantly higher in the HIV-infected compared to the HIV-uninfected PSS group (25.3 pg/mL (IQR, 4.56–41.76) vs 2.73 pg/mL (IQR, 1.55–9.02), *P* = 0.0192). In the HIV-infected PSS group (but not the HIV-infected LS or HIV-uninfected PSS groups) the IP-10, MIP-1b, IL-6 and IL-8 were raised compared to the controls. IL-10 levels decreased but did not return to control baseline values by 6 months in HIV infected PSS and LS and HIV uninfected PSS.

**Conclusion:**

PSS and LS in HIV-infected individuals is characterized by an increase in inflammatory and anti-inflammatory cytokines such as IL-10. The increase of IL-10 is greater in HIV-infected than uninfected individuals. Further work is required to ascertain if this is part of an immunological profile that correlates with adverse outcomes such as serofast syphilis and neurosyphilis, in HIV-infected individuals.

**Electronic supplementary material:**

The online version of this article (doi:10.1186/s12879-017-2201-7) contains supplementary material, which is available to authorized users.

## Background

Syphilis is a multi-stage disease caused by infection with *Treponema pallidum* subsp. *pallidum* (*T. pallidum*). A number of studies have found the natural course of syphilis to be altered in HIV-infected patients. The alterations include a higher rate of asymptomatic primary disease [1], more aggressive primary or secondary stage disease [2, 3] and a higher proportion (up to a quarter) presenting with mixed primary and secondary stage disease at the time of diagnosis [3, 4]. There is also evidence that neurosyphilis may occur more frequently, at an earlier stage and progress more rapidly in the presence of HIV infection [5–7]. Serological cure in the cerebrospinal fluid (CSF) [8] and peripheral blood [3, 9–11] has been shown to be slower in HIV-infected individuals. Unusual serological diagnostic test results such as false negative treponemal and non-treponemal tests in syphilis may also be more common among persons with HIV infection [12, 13].

These findings generate the hypothesis that the immune responses to syphilis differ by HIV-infection status. Most previous studies have characterized the immune response to syphilis in animal models or in HIV-uninfected individuals [14–20]. The only study that we are aware of that has included an HIV-infected population was weakened by its retrospective study design, small sample size, small number of cytokines evaluated and lack of HIV-uninfected and healthy HIV-infected control groups [21].

Establishing if there is a differential immune response to *T. pallidum* infection by HIV status, and more fully characterizing what this response is in HIV-infected individuals, is important for a number of reasons. Firstly, in countries where the incidence of syphilis is increasing, a large proportion of syphilis infections are occurring in HIV-infected persons [22–24]. Typically, an increasing proportion of cases occur in individuals with repeat syphilis [22, 25]. This repeat syphilis is often asymptomatic and diagnosed purely on the basis of increased titres of non-treponemal tests [22, 26]. Since a wide array of other factors can cause changes in these titres, a proportion of these diagnoses of repeat syphilis may be false positives [22, 25]. If a sufficiently distinctive immunological profile of initial and repeat syphilis infection could be established, then this could be used in verifying the diagnosis of syphilis in these reinfection cases. Given the fact that in some populations a majority of syphilis reinfections are in HIV-infected individuals, an important first step in this process is determining what the immune response to syphilis is in HIV-infected versus-uninfected individuals [22]. Secondly, if immune response to syphilis was found to be subverted in HIV then this would pave the way for studies to investigate if this was causally linked to certain poor outcomes in syphilis, such as early neurosyphilis [7].

## Methods

The Institutional Review Board of the Institute of Tropical Medicine (ITM) and the Ethics Committee of the University Hospital Antwerp approved this sub-study (13/44/426). Between May 2014 and June 2015, participants were recruited in the main study “*Treponema pallidum*-specific Proteomic Changes in Patients With Incident Syphilis Infection (SeTPAT)” study (ClinicalTrials.gov Registration Number: NCT02059525) conducted at the ITM in Antwerp, Belgium. All patients attending the ITM, Antwerp’s STI or HIV clinics, over the age of 17 years in whom a new diagnosis of syphilis was made and had not received antibiotics in the preceding 30 days were prospectively recruited into the study (See Additional file 1: Figure S1 for Participant Inclusion Process). The diagnosis and staging of syphilis was according to the Centers for Disease Control and Prevention classification [27]. All patients were assessed according to a standardized protocol that collected detailed information about sexual behavior, clinical features and laboratory tests including Rapid Plasma Reagin test (RPR), *Treponema pallidum* Particle Agglutination (TPPA) and C-Reactive Protein (CRP). The RPR titre was tested using Macro-Vue RPR card tests (Becton Dickinson, Sparks, MD, USA) and the TPPA with SERODIA-TPPA (Fujirebio Inc., Tokyo, Japan), according to the manufacturer’s instructions. HIV viral loads below the limit of detection (20 copies/mL) were given the value of 10 copies/mL. The presence of concomitant sexually transmitted infections (STIs) (gonorrhea and chlamydia) was assessed via an Abbott Real*Time* CT/NG assay (Abbott Molecular, Des Plaines, IL, USA).

Patients were followed up at 3, 6, 9 and 12 months. Patient clinical and laboratory characteristics were recorded at each consultation. In addition, 30 HIV positive controls attending the same HIV clinic and at the same time as the cases were recruited. Blood was drawn into EDTA-coated tubes (Sarstedt Monovette, Nümbrecht, Germany) and separated by centrifugation at 2000 g for 10 min at ambient temperature. Within 3 h of collection, plasma was frozen and stored at −80 °C until testing. To avoid collection bias all samples were processed in the same fashion according to the study standard operating procedures.

Chemokines (Monocyte Chemoattractant Protein [MCP]-1, Macrophage Inflammatory Protein [MIP]-1α, MIP-1β, IFNγ-Inducible Protein [IP]-10, Interleukin [IL]-8) and cytokines (Interferon [IFN]α, IFNγ, IL-1 β, IL-12p40, IL-12p70, IL-4, IL-5, IL-6, IL-7, IL-10 and IL-17A) from the baseline and 6 month post-treatment time-points, and from an additional two individuals 3 and 12 months before their syphilis diagnosis, were measured in a single experiment using a magnetic bead Milliplex™ Human Cytokine kit (Millipore, MA, USA) on a Bio-Plex™ Suspension Array Reader (Bio-Rad Laboratories Inc®, CA, USA) in accordance with the manufacturer’s instructions. Standard curves were constructed using duplicate measurements of kit standards. Samples below the limit of quantification were assigned the value of half the lowest limit detected for each cytokine (see Additional file 1: Table S1).

### Statistical analyses

Values are summarized as medians and interquartile ranges (IQR). In keeping with previous studies, we found that the immunological profiles of syphilis differed between primary and secondary stage syphilis combined group (PSS) and early and late latent stage syphilis combined group (LS) [20, 21]. All results are therefore presented stratified by PSS versus LS. There were only two HIV-uninfected patients diagnosed with LS and no comparisons are therefore made with this group. Comparison of the inter-individual data between the PSS, and LS groups was performed using the Mann-Whitney *U*-test. Wilcoxon signed rank test was used to evaluate intra-individual changes from baseline to six-month visit. Correlations were assessed via Spearman’s rank correlation coefficient. Due to the small sample size and the fact that this is the first study of its kind, the study was conceived as being exploratory rather than hypothesis testing. This, combined with the fact that the cyto- and chemokines under investigation are known to be inter-dependent, led us to not utilize Bonferroni corrections [28–30]. All analyses were performed in Stata 13 (StataCorp LP, College Station, TX, USA). A *P*-value of less than 0.05 was considered statistically significant.

## Results

A total of 79 HIV-infected [44 PSS and 35 LS] and 12 HIV-uninfected (10 PSS and 2 LS) cases of syphilis and 30 HIV-infected controls were included in the study (see Additional file 1: Figure S1 for inclusion process). At baseline, the age, number of sex partners in the prior 12 months, proportion with a previous episode of treated syphilis, prevalence of other STIs diagnosed at baseline, RPR titre and syphilis treatment received did not differ significantly between HIV-infected and uninfected PSS groups (Table 1). Likewise, except for a higher HIV viral load in the controls than the PSS and LS groups, there was no significant difference in the age, CD4 T cell count, percent on antiretroviral therapy (ART) or HIV viral load between the controls and HIV-infected syphilis patients. All patients were treated with intramuscular benzathine penicillin G, except for two patients who were treated with oral doxycycline. Only one participant was a woman. She was HIV negative and presented with secondary syphilis. All the men with syphilis, excluding two individuals, reported being men who have sex with men (MSM).

**Table 1:** Baseline characteristics

	Controls	Primary/Secondary syphilis			Latent Syphilis
	(*n* = 30)	HIV+ (*n* = 44)^#^	HIV-(*n* = 10)^#^	HIV+ vs. HIV-, P^$^	HIV+(*n* = 35)^#^
Men	30 (100%)	44 (100%)	9 (90%)*	0.0359	35 (100%)
Age (years)	37 (32–45)	39.5 (30.5–50.0)	34 (30–43)	0.4689	40 (35–46)
MSM	24 (80%)	44 (100%)	9 (90%)	0.0359	34 (94.7.1%)
CD4 T cell count (cells per μL)	577 (392–684)	649 (452–838)	800 (800–800)	0.4537	590 (433–691)
HIV Viral Load (copies per mL)	34 (10–814)	10 (10–53)*	NA	NA	10 (10–25)**
On Antiretroviral Therapy	24 (80%)	39 (88.6%)	NA	NA	31 (88.6%)
RPR titre	0 (0–0)	1/64 (1/32–1/128)	1/64 (1/16–1/128)	0.5556	1/64 (1/16–1/128)
Treatment				1.0	
Benzathine-penicillin G	NA	44 (100%)	10 (100%)		33 (94.3%)
Doxycycline	NA	0	0		2 (5.7%)
Previous treated syphilis	NA	27 (61.3%)	3 (30%)	0.0743	24 (68.6%)
Other STIs present^a^	0 (30)	1 (2.3%)	0 (0%)	0.63	2 (5.7%)
No. of sex partners prior 12 months	1.5 (1–6)	8.5 (2–26)**	6.5 (2–8)*	0.5174	7 (1–12)*

Data are n (%), median (IQR), unless otherwise stated. NA - Not Applicable

*P < 0.05, ** *P* < 0.005

^#^ *P*-value is for comparison with controls at baseline (Mann-Whitney *U*-test, excluding ‘Men’, ‘MSM’, ‘On Antiretroviral Therapy’ and ‘Other STIs’ where Fisher’s exact test used)

^$^ *P*-value is for comparison between HIV-infected and uninfected groups (Mann-Whitney *U*-test, except ‘Men’, ‘MSM’, ‘Treatment’, ‘Previous treated syphilis’ and ‘Other STIs’ where Fisher’s exact test used)

^a^Presence of *N. gonorrhoeae* or *C. trachomatis* in urethra, rectum or oropharynx

MSM: men who have sex with men

### Baseline immunological profile

**Fig. 1:**
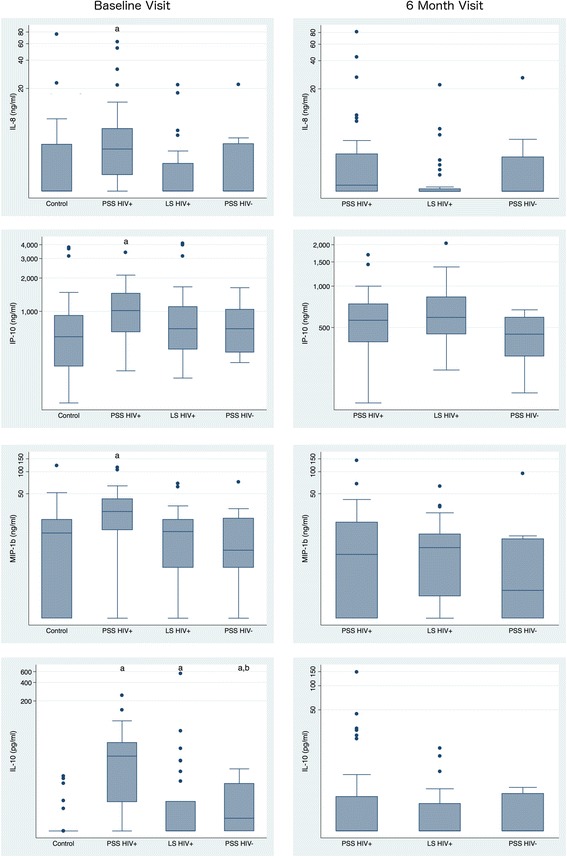
Plasma cytokine and chemokine concentrations in HIV-infected and uninfected patients with syphilis by syphilis stage at baseline and 6 month visit. Cytokine levels in each group (pg/ml, Y axis log scale) are shown as median and interquartile range (box), with 10th and 90th percentiles (whiskers). Letters (**a**) and (**b**) above the box-plot indicate statistically significant differences (*P* < 0.05) as compared with the HIV-infected controls and the comparison between HIV-infected and uninfected PSS groups, respectively. Abbreviations. PSS HIV+: Primary Secondary Syphilis HIV-infected, LS HIV+: Latent Syphilis HIV-infected, PSS HIV-: Primary Secondary Syphilis HIV-uninfected

At the baseline visit, compared to the control group, concentrations of the anti-inflammatory cytokine IL-10 were significantly elevated in both the HIV-infected and uninfected groups (Fig. 1; Table 2). The level of IL-10 was strikingly higher in the HIV-infected PSS compared to the HIV-uninfected PSS group (25.3 pg/mL (IQR, 4.56–41.76) versus 2.73 pg/mL (IQR, 1.55–9.02), *P* = 0.0192). In the HIV-infected PSS group (but not the HIV-infected LS or HIV-uninfected PSS groups) the chemokines IP-10, MIP-1β, IL-8 and the pro-inflammatory cytokine IL-6 were raised significantly compared to the controls. Of these only the MIP-1β level was significantly higher in the HIV-infected PSS than uninfected PSS group (*P* = 0.0205).

**Table 2:** Plasma cytokine and chemokine concentrations in HIV-infected and uninfected patients with syphilis by syphilis stage (pg/ml)

	HIV-infected Controls	HIV-infected		HIV-uninfected	
		Primary/Secondary Syphilis^#^	Latent Syphilis^#^	Primary/Secondary Syphilis^#^	PSS HIV+ vs. -, P^$^
Baseline					
N	30	44	35	10	
Pro-inflammatory					
IFNα	9.88 (1.6–16.43)	7.45 (0.8–31.35)	7.45 (0.8–20.42)	4.12 (0.8–24.73)	0.5202
IL1β	1.57 (1.57–1.57)	1.57 (1.57–1.57)	1.57 (1.57–1.57)	1.57 (1.57–1.57)	0.4883
IL-6	1.26 (1.26–1.26)	1.26 (1.26–1.26)*	1.26 (1.26–1.26)	1.26 (1.26–1.26)	0.722
IL-17A	1.52 (1.52–1.52)	1.52 (1.52–1.52)	1.52 (1.52–1.52)	1.52 (1.52–1.52)	0.8358
Th1					
IFNγ	2.18 (1.54–4.59)	3.52 (1.54–5.45)	1.79 (1.54–4.59)	4.01 (1.54–16.66)	0.5991
IL-7	1.11 (1.11–1.11)	1.11 (1.11–1.36)	1.11 (1.11–1.11)	1.11 (1.11–1.11)	0.357
IL-12p40	1.02 (1.02–1.02)	1.02 (1.02–1.02)	1.02 (1.02–1.02)	1.02 (1.02–1.02)	0.4056
IL-12p70	1.61 (1.61–1.61)	1.61 (1.61–1.67)	1.61 (1.61–1.61)	1.61 (1.61–4.08)	0.6264
Chemokines					
IL-8	1.6 (1.6–5.06)	4.51 (2.41–7.43)**	1.6 (1.6–3.15)	1.6 (1.6–5.13)	0.0723
IP-10	589.4 (319.38–917.05)	1016.1 (649.64–1451.32)**	696.89 (455.35–1101.67)	696.52 (426.27–1040.07)	0.1561
MCP-1	258.42 (179.09–307.75)	260.1 (216.95–307.35)	242.05 (180.35–303.1)	247.64 (205.76–287.32)	0.4321
MIP-1α	1.63 (1.63–3.65)	3.09 (1.63–8.3)	1.63 (1.63–1.63)	1.63 (1.63–1.63)	0.1247
MIP-1β	14.54 (0.99–22.23)	28.59 (16.06–42.51)**	15.2 (4.92–22.23)	8.46 (4.92–23.05)	0.0205
Th2					
IL-4	1.58 (1.58–1.58)	1.58 (1.58–1.58)	1.58 (1.58–1.58)	1.58 (1.58–1.58)	0.4883
IL-5	1.58 (1.58–1.58)	1.58 (1.58–1.58)	1.58 (1.58–1.58)	1.58 (1.58–1.58)	0.3324
Anti-inflammatory					
IL-10	1.55 (1.55–1.55)	25.3 (4.56–41.76)***	2.46 (1.55–4.62)*	2.73 (1.55–9.02)*	0.0029
6 month					
N		41	34	10	
Pro-inflammatory					
IFNα		0.8 (0.8–9.27)**^#^	0.8 (0.8–7.8)**^#^	0.8 (0.8–0.8)*^#^	0.3282
IL-1β		1.57 (1.57–1.57)	1.57 (1.57–1.57)	1.57 (1.57–1.57)	0.1798
IL-6		1.26 (1.26–1.26)	1.26 (1.26–1.26)	1.26 (1.26–1.26)	0.7379
IL-17A		1.52 (1.52–1.52)	1.52 (1.52–1.52)	1.52 (1.52–1.52)	0.7379
Th1					
IFNγ		1.54 (1.54–5.09)	1.62 (1.54–4.88)	5.17 (1.54–13.89)	0.0978
IL-7		1.11 (1.11–1.11)	1.11 (1.11–1.11)	1.11 (1.11–1.11)	0.6486
IL-12p40		1.02 (1.02–1.02)	1.02 (1.02–1.02)	1.02 (1.02–1.02)	0.2068
IL-12p70		1.61 (1.61–1.61)	1.61 (1.61–1.86)	1.61 (1.61–1.61)	0.7639
Chemokines					
IL-8		1.87 (1.6–4.02)	1.6 (1.6–1.7)	1.66 (1.6–3.75)	0.4406
IP-10		564.56 (392.18–740.03)	592.04 (447.8–831.96)	447 (309–593)	0.2134
MCP-1		270.25 (225.5–330.3)	261.32 (241.06–312.26)	269 (219–297)	0.8287
MIP-1α		1.63 (1.63–1.63)	1.63 (1.63–1.63)	1.63 (1.63–1.63)	0.6726
MIP-1β		7.4 (0.99–20.28)	9.17 (1.99–14)	2.39 (0.99–12.05)	0.249
Th2					
IL-4		1.58 (1.58–1.58)	1.58 (1.58–1.58)	1.58 (1.58–1.58)	0.1798
IL-5		1.58 (1.58–1.58)	1.58 (1.58–1.58)	1.58 (1.58–1.58)	0.3621
Anti-inflammatory					
IL-10		1.83 (1.83–4.09)***	2.11 (1.83–3.37)***	2.26 (1.83–4.46)**	0.6296

All values are median and interquartile range. Data are n (%), median (IQR), unless otherwise stated

^#^ *P*-value is for comparison with controls at baseline (Mann-Whitney *U*-test)

^$^ *P*-value is for comparison between HIV-infected and uninfected groups with Primary/Secondary syphilis (Mann-Whitney *U*-test)

*P < 0.05, ** *P* < 0.005, ***P < 0.0005

PSS - Primary/Secondary Syphilis

### Six-month immunological profile

In seven out of 91 individuals with syphilis we had baseline but no 6-month samples. At 6 months post syphilis treatment the levels of IL-10 had declined in all groups, and most strikingly in the HIV-infected PSS group. IL-10 remained significantly elevated compared to the control group (*P* < 0.0001 to 0.0018). There was no longer a difference in IL-10 levels between the HIV-infected and uninfected PSS groups. Another interesting observation was that IFNα levels declined in all groups and at 6 months attained significantly lower levels than the HIV-infected control group (*P* = 0.0007 to 0.0104).

### Intra-individual change in IL-10 & IFNα concentrations

#### IL-10

**Fig. 2:**
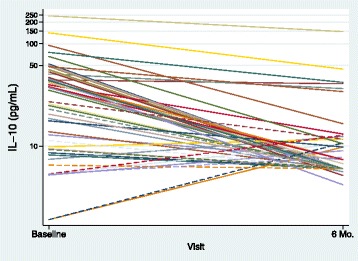
IL-10 response to primary and secondary syphilis infection in HIV-infected (solid lines) and uninfected individuals (dashed lines) at the time of infection (baseline visit) and at 6 months follow up (log scale)

There was a significant decline in IL-10 from baseline to 6 month visit in the HIV-infected PSS group (*P* < 0.0001), and a non-significant decline in the HIV-infected LS group and the HIV-uninfected PSS group (*P* = 0.4803 and 0.4838, respectively; Table 2 & Fig. 2).

#### IFNα

Over the same time period, there was a significant decline in IFNα in the HIV-infected LS group (*P* < 0.0412), but this change was borderline significant in the HIV-infected PSS group (*P* = 0.0568) and not significant in the HIV-uninfected PSS group (*P* = 0.3173; Table 2 & Fig. 1).

In the two individuals for which we were able to test their cytokine profile before, during and after PSS infection there was an increase in IL-10 at the time of infection and a return to pre-syphilis values after 6 months. These individuals were originally included as controls and then during the course of the study developed a syphilis infection. Conversely, IFNα levels in these two decreased at the time of secondary stage syphilis diagnosis and continued to drop until the 6-month visit.

### IL-10 Correlation analyses

At the baseline visit there were differences between the HIV-infected and uninfected groups in the immunological markers that were correlated with IL-10 level. In the HIV-infected PSS group, IL-10 was significantly positively correlated with IL-12p40, IL-12p70, MIP-1α, MIP-1β, IL-5, IL-6, IL-7 and IL-8 (r = 0.36, 0.67, 0.59, 0.61, 0.75, 0.79, 0.83, 0.53, respectively; Additional file 1: Table S2a). In the HIV-uninfected group there were no significant correlations with IL-10 (Additional file 1: Table S2b). There were however only 10 individuals in this group.

None of the cohort was diagnosed with treatment failure or neurosyphilis subsequent to the baseline visit and we were thus unable to assess if any particular immunological profile was predictive of these adverse outcomes.

In sensitivity analyses we excluded the one woman from the analyses and repeated the analyses with the PSS group split into primary syphilis (PS) and secondary syphilis (SS) and the LS group split into early latent and late latent syphilis. We also repeated the analyses with the HIV-infected group limited to those taking ART. These made little difference to the results (results not shown). Scatterplots revealed that the difference in IL-6 between controls and the HIV-infected PSS group was largely driven by two outlier values. Repeating the comparison between these two groups excluding these two outliers, there was no longer a statistically higher IL-6 level in the HIV-infected group.

## Discussion

The most prominent difference in the plasma immunological response to PSS between the HIV-infected and uninfected was a ten-fold higher IL-10 response in the HIV positive group. Although this is a new finding, it is not incommensurate with findings from other studies. The study by Knudsen et al. [21] found similarly high levels of IL-10 in HIV-infected PSS (46.7 pg/mL; IQR 28.4–78.9) and LS (13.4 pg/mL; IQR 6.5–31.1). Many studies in HIV-uninfected populations have not assessed IL-10 levels [15, 17, 31]. Those that have, found either IL-10 to be not raised [14, 19] or marginally raised [16, 20] compared to HIV-uninfected controls. These studies used differing methodologies and as a result IL-10 values cannot be meaningfully compared between studies. Our study builds on these results by for the first time comparing immunological profiles in HIV-infected and uninfected groups.

### How should we interpret the higher IL-10 levels in the HIV-infected individuals and in the PSS group in particular?

The initial immune response to *T. pallidum* involves a robust cell mediated response characterized by a Th1 predominance that results in the clearance of the vast majority of treponemes at the lesion of the primary stage [15, 32]. We mainly found increased levels of IL-10 and chemokines in the PSS group. The positive correlations we found between IL-10 and IL-12p40, IL-12p70, MIP-1α, MIP-1β, IL-5, IL-6, IL-7 and IL-8 in the HIV-infected PSS group could be interpreted as evidence that the increased IL-10 is acting as a feedback response to limit inflammation. We did not see any particular increase of Th1 cytokines as reported by two other studies [15, 29].

An alternative/complementary explanation is that *T. pallidum* is playing a role in the IL-10 elevation. In a proportion of cases, *T. pallidum* is able to evade the immune system and set up secondary and chronic latent stages of infection [14]. Poor antigenicity and antigenic variation have been shown to play an important role in this regard [33–35]. A further mechanism is that an enhanced regulatory T-cell (Treg) response in early syphilis may down-regulate the immune response to *T. pallidum* and thereby facilitate its survival [17, 31, 36]. IL-10 plays an important role in the establishment of the Treg anti-inflammatory effect that is important in preventing overwhelming inflammatory responses [37]. These immunosuppressive properties of IL-10 have also been shown to be harnessed by a number of pathogens to facilitate persistent infection: *Borrelia burgdorferi* [38], *Plasmodium* spp. [39], *Leishmania* spp. [40] and *Mycobacterium tuberculosis* [37].

Babolin et al. provided experimental evidence that the *T. pallidum* protein, TpF1 bacterioferrin, is able to induce a Treg response in patients with secondary syphilis via the induction of IL-10 and TGF-β [41]. Podwinska and colleagues investigated the ability of lymphocytes to produce cytokines from patients at different stages of syphilis in response to stimulation with *T. pallidum* antigen [20]. They noted a stepwise increase in IL-10 production from primary syphilis to secondary and latent syphilis that correlated inversely with the ability to produce IL-2 (a Th1 cytokine). In a study of 531 syphilis patients with and without neurosyphilis, Li and colleagues found evidence that Tregs play a role in treponemal persistence and the genesis of neurosyphilis in HIV-uninfected individuals [17]. They demonstrated that secondary and serofast syphilis patients had increased Treg percentages in their peripheral blood compared with healthy controls. Patients with neurosyphilis had a higher frequency of Tregs in peripheral blood compared to non-neurosyphilis patients. In a similar vein, Pastuszczak et al.*,* found that IL-10 was considerably higher in the CSF of patients with neurosyphilis than syphilis patients without neurological involvement [19]. These findings are also consistent with the clinical study by Knudsen et al.*,* which was able to demonstrate with a high degree of probability that *T. pallidum* infection was the cause of the IL-10 elevation [21].

HIV-infection is characterized by a Th1 to Th2 cytokine shift during the course of the infection [42] which includes an enhanced potential for robust IL-10 and associated Treg response [43–45]. If HIV infection leads to a more pronounced Treg response to infection with *T. pallidum,* then this could explain a number of the atypical features of syphilis in HIV-infected individuals mentioned in the introduction. The recently established correlation between Treg activity and risk of serofast syphilis and neurosyphilis [17] in HIV-uninfected individuals suggests the need for more investigations along these lines in HIV co-infected patients. The discovery of an immunological profile strongly associated with asymptomatic neurosyphilis would be of considerable clinical utility.

Limitations in our study include that we only measured systemic cytokine responses and not cytokine secretions in response to antigenic (*T. pallidum* antigen) stimulation. The detection of cytokines in plasma samples has several limitations. First of all, cytokine responses are mainly local and short-ranged. We only detect cytokine overflow in the systemic circulation. Secondly, we have no data about the cell types that secreted these and conclusions are thus largely speculative. Thirdly, cytokine measurements in plasma are critically dependent on proper sample collection, storage and the technology used to detect them. We ensured that the cytokine detection was done under optimal circumstances such as prompt sample processing in order to minimize undesirable test variation due to suboptimal storage and test conditions.

In addition, the small sample size and the fact that almost the entire cohort were MSM, with high-risk behavior, thereby reducing the generalizability of the results. We did not resample the control group at 6-months and thus did not have a control group for the 6-month samples. The fact that those diagnosed with syphilis reported a higher number of sexual partners than the controls raises the possibility that they were co-infected with other undiagnosed STIs. Given the fact that there was no statistically significant difference in the number of partners, or the diagnosis of STIs between the PSS HIV-infected and uninfected subjects, this explanation is unlikely to explain the immunological differences found between these two groups. Our HIV-infected patients included both those receiving and not receiving ART, which may have influenced our results, however, we consider this as unlikely as repeating the analyses restricted to those on ART did not substantively change the results. We did not assess patients’ genotypes for IL-10 and other cytokines. A number of IL-10 single nucleotide polymorphisms have been found to be associated with both IL-10 inducibility [19] and susceptibility to infectious diseases such as tuberculosis [46] and neurosyphilis [19]. For a number of the chemo- and cytokines, only a small proportion of samples had levels above the level of detection (Additional file 1: Table S1), particularly in the cases of IL1b, IL-6, IL-17A, IL-7, IL-12p40, IL-4 and IL-5. A number of these low abundance chemo- and cytokines had low correlations between technical duplicates (Additional file 1: Table S1). The results for these chemo- and cytokines need to be interpreted with considerable caution. Furthermore, we did not correct for multiple comparisons which may have led to type I errors [28]. The strengths of the study include its setting within a prospective observational cohort study wherein the patients had their behavioral, clinical and laboratory characteristics collected in a detailed and standardized fashion.

## Conclusion

In conclusion, we demonstrate that PSS and LS in HIV-infected individuals is characterized by not only an increase in inflammatory- but also the anti-inflammatory-cytokine, IL-10. The increase of IL-10 is greater in HIV-infected than uninfected individuals and declines but does not return to baseline levels by 6-month post- infection. Further work is required to ascertain if there is an immunological profile that specifically correlates with adverse outcomes such as treatment failure, serofast- and neurosyphilis in HIV-co-infected individuals.

## Additional file

Additional file 1: Table S1. Cytokine data quality assessment. **Table S2.a** Correlations between cytokines/chemokines in primary/secondary syphilis in HIV-infected individuals at baseline. **Table S2.b** Correlations between cytokines/chemokines in primary/secondary syphilis in HIV-uninfected individuals at baseline. (DOCX 51 kb)

## Abbreviations

ART: Antiretroviral therapy
CRP: C-Reactive Protein
CSF: Cerebrospinal fluid
HIV: Human Immunodeficiency Virus
IL: Interleukin
IQR: Interquartile range
ITM: Institute of Tropical Medicine
LS: Latent Syphilis
MCP: Monocyte Chemoattractant Protein
MIP: Macrophage Inflammatory Protein
MSM: Men who have sex with men
PS: Primary Syphilis
PSS: Primary/Secondary Syphilis
RPR: Rapid Plasma Reagin test
SS: Secondary Syphilis
STI: Sexually Transmitted Infection
TPPA: *Treponema pallidum* Particle Agglutination
