# Supplementary material for: The immunological response to syphilis differs by HIV status; a prospective observational cohort study
Source: BMC Infect Dis. 2017 Jan 31;17:111. doi: 10.1186/s12879-017-2201-7 (PMC5286814; doi:10.1186/s12879-017-2201-7)
Supplement: Additional file 1: Table S1. — Cytokine data quality assessment. Table S2.a Correlations between cytokines/chemokines in primary/secondary syphilis in HIV-infected individuals at baseline. Table S2.b Correlations between cytokines/chemokines in primary/secondary syphilis in HIV-uninfected individuals at baseline. (DOCX 51 kb) [file 12879_2017_2201_MOESM1_ESM.docx]

**Supplementary Information**

Supplementary Figure 1. Flow chart showing participant recruitment

167 screened

30 controls

12 HIV uninfected

79 HIV infected

91 syphilis cases

120 syphilis cases

150 included

17 not enrolled

10 patients with history of antibiotic use last month

1 alternative diagnosis (RPR negative, non-syphilis diagnosis)

3 declined participation

1 control patient HIV-negative

2 patients already enrolled in the study

29 excluded due to insufficient plasma sample available

Supplementary Table 1. Cytokine and chemokine data quality assessment.

| Cytokine | CV values of duplicate samples with detectible values  (N=detectable sample(s)/ CV%) | Correlation of subset (N=15) of samples tested in duplicate ^$^ | P-value | Samples with undetectable concentrations (%) | Lowest level of detection (pg/mL) |
| --- | --- | --- | --- | --- | --- |
| IFNα | 5/ 37.9 | 0.81 | 0.0005 | 49.5 | 1.6 |
| IL1β | # | 0.57 | 0.0342 | 96.3 | 3.1 |
| IL-6 | # | #^#^ | - | 92.2 | 2.5 |
| IL-17A | 5/ 14.7 | 0.67 | 0.0004 | 90.4 | 3.1 |
| IFNγ | 5/ 37.9 | 0.93 | <0.0001 | 46.3 | 3.1 |
| IL-7 | # | # | 0.0093 | 86.2 | 2.2 |
| IL-12p40 | 1/ 20.3 | 1.0 | <0.0001 | 95.8 | 2.1 |
| IL-12p70 | 3/ 31.3 | 0.62 | 0.0187 | 78.9 | 3.2 |
| IL-8 | 4/ 4.2 | 0.88 | <0.0001 | 46.3 | 3.2 |
| IP-10 | 15/ 2.3 | 1.0 | <0.0001 | 0 | 3.9 |
| MCP-1 | 15/ 3.2 | 1.0 | <0.0001 | 0 | 0.4 |
| MIP-1α | 2/ 18.2 | 0.77 | 0.0706 | 72.5 | 3.3 |
| MIP-1β | 9/ 19.8 | 0.77 | 0.0012 | 22.9 | 2.0 |
| IL-4 | # | #^#^ | - | 96.8 | 3.2 |
| IL-5 | # | #^#^ | - | 90.4 | 3.2 |
| IL-10 | 5/ 8.1 | 0.99 | <0.0001 | 57.2 | 3.1 |

^$^ Correlations of duplicates tested using Pearson’s correlation

^#^ Values for all duplicate samples were below the level of detection

**Supplementary Table 2a.** Correlations between cytokines/chemokines in primary/secondary syphilis in HIV-infected individuals at baseline (**P* <0.05)

|  | IFNα | IFNγ | IL-1β | IL-12p40 | IL-12p70 | IP-10 | MCP-1 | MIP-1α | MIP-1β | IL-4 | IL-5 | IL-6 | IL-7 | IL-8 | IL-10 | IL-17A | CRP |
| --- | --- | --- | --- | --- | --- | --- | --- | --- | --- | --- | --- | --- | --- | --- | --- | --- | --- |
| IFNα | 1 |  |  |  |  |  |  |  |  |  |  |  |  |  |  |  |  |
| IFNγ | 0.4077* | 1 |  |  |  |  |  |  |  |  |  |  |  |  |  |  |  |
| IL-1b | 0.3841* | 0.8194* | 1 |  |  |  |  |  |  |  |  |  |  |  |  |  |  |
| IL-12p40 | 0.3634* | 0.1616 | 0.1706 | 1 |  |  |  |  |  |  |  |  |  |  |  |  |  |
| IL-12p70 | 0.3635* | 0.3159* | 0.3544* | 0.4410* | 1 |  |  |  |  |  |  |  |  |  |  |  |  |
| IP-10 | -0.0139 | 0.1333 | 0.0883 | -0.1273 | -0.1384 | 1 |  |  |  |  |  |  |  |  |  |  |  |
| MCP-1 | -0.142 | 0.0777 | 0.0127 | -0.2642 | -0.0984 | 0.2645 | 1 |  |  |  |  |  |  |  |  |  |  |
| MIP-1α | 0.3730* | 0.2574 | 0.1319 | 0.7887* | 0.5356* | -0.0791 | -0.0611 | 1 |  |  |  |  |  |  |  |  |  |
| MIP-1β | 0.2743 | 0.1029 | 0.0171 | 0.6892* | 0.4440* | 0.0295 | -0.0644 | 0.8387* | 1 |  |  |  |  |  |  |  |  |
| IL-4 | 0.3392* | 0.8706* | 0.9737* | 0.141 | 0.3029* | 0.1223 | 0.0788 | 0.1283 | 0.0357 | 1 |  |  |  |  |  |  |  |
| IL-5 | 0.3188* | 0.114 | -0.0068 | 0.7802* | 0.7285* | -0.1305 | -0.2295 | 0.8189* | 0.6900* | -0.0508 | 1 |  |  |  |  |  |  |
| IL-6 | 0.2281 | 0.1238 | 0.0855 | 0.4131* | 0.9366* | -0.108 | -0.0893 | 0.5407* | 0.4942* | 0.0507 | 0.8067* | 1 |  |  |  |  |  |
| IL-7 | 0.3595* | 0.3029* | 0.201 | 0.4296* | 0.8837* | -0.0635 | -0.1006 | 0.5945* | 0.4808* | 0.1471 | 0.8201* | 0.9300* | 1 |  |  |  |  |
| IL-8 | 0.2503 | 0.0233 | -0.0337 | 0.8432* | 0.5154* | -0.0503 | -0.1525 | 0.8787* | 0.7658* | -0.0553 | 0.8756* | 0.5607* | 0.5386* | 1 |  |  |  |
| IL-10 | 0.2273 | 0.1054 | -0.0694 | 0.3622* | 0.6655* | 0.0771 | 0.0251 | 0.5848* | 0.6109* | -0.1075 | 0.7475* | 0.7941* | 0.8285* | 0.5334* | 1 |  |  |
| IL-17A | -0.104 | 0.02 | 0.0412 | -0.0283 | -0.0174 | 0.0339 | -0.0497 | -0.0802 | 0.0119 | 0.0416 | -0.0344 | -0.0218 | -0.0232 | 0.0445 | 0.0809 | 1 |  |
| CRP | -0.2955 | -0.0598 | -0.1185 | -0.1828 | -0.2526 | 0.0114 | 0.1622 | -0.0839 | 0.1435 | -0.035 | -0.2306 | -0.1976 | -0.2457 | -0.1067 | 0.0171 | 0.4592* | 1 |

**Supplementary table 2b.** Correlations between cytokines/chemokines in primary/secondary syphilis in HIV-uninfected individuals at baseline (* P <0.05)

|  | IFNα | IFNγ | IL1β | IL-12p70 | IP-10 | MCP-1 | MIP-1α | MIP-1β | IL-4 | IL-6 | IL-7 | IL-8 | IL-10 | IL-17A | CRP |
| --- | --- | --- | --- | --- | --- | --- | --- | --- | --- | --- | --- | --- | --- | --- | --- |
| IFNα | 1 |  |  |  |  |  |  |  |  |  |  |  |  |  |  |
| IFNγ | 0.8480* | 1 |  |  |  |  |  |  |  |  |  |  |  |  |  |
| IL-1β | 0.7801* | 0.8449* | 1 |  |  |  |  |  |  |  |  |  |  |  |  |
| IL-12p70 | 0.8379* | 0.8546* | 0.9940* | 1 |  |  |  |  |  |  |  |  |  |  |  |
| IP-10 | -0.1121 | 0.1735 | -0.0725 | -0.0937 | 1 |  |  |  |  |  |  |  |  |  |  |
| MCP-1 | 0.1216 | 0.1559 | 0.1928 | 0.1941 | 0.4052 | 1 |  |  |  |  |  |  |  |  |  |
| MIP-1α | 0.8357* | 0.9358* | 0.9661* | 0.9693* | -0.0106 | 0.1265 | 1 |  |  |  |  |  |  |  |  |
| MIP-1β | 0.7957* | 0.7534* | 0.8961* | 0.9133* | -0.0251 | 0.0826 | 0.8754* | 1 |  |  |  |  |  |  |  |
| IL-4 | 0.7801* | 0.8449* | 1.0000* | 0.9940* | -0.0725 | 0.1928 | 0.9661* | 0.8961* | 1 |  |  |  |  |  |  |
| IL-6 | 0.7801* | 0.8449* | 1.0000* | 0.9940* | -0.0725 | 0.1928 | 0.9661* | 0.8961* | 1.0000* | 1 |  |  |  |  |  |
| IL-7 | 0.7801* | 0.8449* | 1.0000* | 0.9940* | -0.0725 | 0.1928 | 0.9661* | 0.8961* | 1.0000* | 1.0000* | 1 |  |  |  |  |
| IL-8 | 0.8781* | 0.8580* | 0.9605* | 0.9793* | -0.0791 | 0.1424 | 0.9616* | 0.9623* | 0.9605* | 0.9605* | 0.9605* | 1 |  |  |  |
| IL-10 | 0.179 | -0.1784 | -0.2504 | -0.1675 | -0.2807 | -0.0254 | -0.1838 | -0.1103 | -0.2504 | -0.2504 | -0.2504 | -0.0783 | 1 |  |  |
| IL-17A | 0.7801* | 0.8449* | 1.0000* | 0.9940* | -0.0725 | 0.1928 | 0.9661* | 0.8961* | 1.0000* | 1.0000* | 1.0000* | 0.9605* | -0.2504 | 1 |  |
| CRP | 0.2541 | 0.1763 | -0.0913 | -0.0647 | -0.2307 | -0.412 | -0.0168 | -0.082 | -0.0913 | -0.0913 | -0.0913 | -0.039 | 0.277 | -0.0913 | 1 |
